# Incremental diagnostic value of coronary CT angiography-derived pericoronary adipose tissue radiomics for identifying hemodynamically significant coronary stenosis

**DOI:** 10.3389/fcvm.2026.1943961

**Published:** 2026-09-29

**Authors:** Wei Fan, Hong-Xuan Wang, Bai-Hui Zhang, Chao Zheng, Xuan Nie, Yang Liu, Bing-Qiang Xu, Yan Zhang, Hong Zhang, Ming-Gang Huang

**Affiliations:** 1 Medical Imaging Center, Shaanxi Provincial People’s Hospital, Xi’an, China; 2 Lintong Rehabilitation and Convalescent Center of PLA Joint Logistic Support Force, Xi’an, China; 3 Shukun Beijing Technology Co., Ltd., Beijing, China; 4 Northwestern Polytechnical University, Xi’an, China; 5 Shaanxi Qian Yichuan Information Technology Co., Ltd., Xi’an, China; 6 Department of Vascular Surgery, Shaanxi Provincial People’s Hospital, Xi’an, China

**Keywords:** coronary artery stenosis, coronary computed tomography angiography, fractional flow reserve, machine learning, pericoronary adipose tissue, radiomics

## Abstract

**Background:**

Pericoronary adipose tissue (PCAT) radiomics may complement coronary computed tomography angiography (CCTA)-based anatomical and physiological assessment. This study aimed to evaluate its incremental diagnostic value for identifying hemodynamically significant coronary stenosis.

**Methods:**

This retrospective study screened 21,438 consecutive patients and included 81 patients with 107 target vessels who underwent CCTA, invasive coronary angiography, and invasive fractional flow reserve (FFR) measurement within 30 days. Patients were allocated at the patient level to training and validation cohorts in a 7:3 ratio. A radiomics score (Radscore) was developed from lesion-specific PCAT features using training-only preprocessing and least absolute shrinkage and selection operator logistic regression. Models incorporating diameter stenosis (DS), CCTA-derived FFR (CT-FFR), fat attenuation index (FAI), and Radscore were evaluated against invasive FFR ≤0.80 using patient-cluster bootstrap inference.

**Results:**

The training and validation cohorts comprised 57 and 24 patients with 75 and 32 vessels, respectively, without patient overlap. In validation, Radscore alone had an area under the receiver operating characteristic curve (AUC) of 0.684 (95% confidence interval [CI], 0.451–0.873). CT-FFR+ Radscore showed higher discrimination than CT-FFR alone (AUC, 0.879 vs. 0.762; difference, 0.117; 95% CI, 0.005–0.229; *P* = 0.041), as did DS+ Radscore vs. DS alone (AUC, 0.812 vs. 0.619; difference, 0.193; 95% CI, 0.018–0.367; *P* = 0.030). The FAI comparison was not significant (*P* = 0.238). At training-derived cutoffs, CT-FFR+ Radscore had lower sensitivity (50.0% vs. 78.6%) and negative predictive value (69.6% vs. 81.3%) but higher specificity (88.9% vs. 72.2%).

**Conclusions:**

Adding Radscore was associated with higher discrimination for CT-FFR and DS in internal validation but did not uniformly improve threshold-dependent classification. Independent external validation is warranted.

## 1 Introduction

Ischemic heart disease remains a leading cause of mortality and disease burden worldwide, with coronary atherosclerosis representing its principal pathological substrate (1, 2). Accurate identification of coronary stenoses associated with myocardial ischemia is central to the management of patients with suspected or known coronary artery disease (CAD), because treatment decisions depend on physiological relevance as well as anatomical severity (3). Invasive fractional flow reserve (FFR) is the reference standard for lesion-specific physiological assessment and quantifies the pressure gradient across an epicardial stenosis during hyperemia (4, 5). However, its invasive nature, requirement for pressure-wire instrumentation and hyperemic agents, procedural cost, and associated risks limit its routine use as a first-line test (6).

Coronary computed tomography angiography (CCTA) is an established noninvasive modality for evaluating CAD and excluding obstructive coronary lesions (7, 8). Nevertheless, diameter stenosis (DS) has limited specificity for identifying ischemia-producing lesions because anatomical severity does not always correspond to physiological significance (9). Computed tomography-derived FFR (CT-FFR) provides a noninvasive estimate of lesion-specific physiology from standard CCTA data, but its performance may be affected by image quality, coronary segmentation, calcification, and computational assumptions (10, 11). Beyond luminal anatomy and pressure-derived indices, pericoronary adipose tissue (PCAT) may reflect local vascular inflammatory signaling and alterations in the coronary microenvironment (12, 13). The fat attenuation index (FAI) summarizes mean PCAT attenuation and has been associated with plaque characteristics and functional lesion significance (14, 15). Radiomics extends this assessment by quantifying intensity distributions, spatial heterogeneity, and texture patterns that are not captured by mean attenuation alone (16). Whether lesion-specific PCAT radiomics provides complementary discrimination beyond DS, CT-FFR, and conventional FAI therefore warrants further evaluation.

Previous studies have suggested that PCAT radiomics may provide complementary information for identifying functionally significant coronary stenosis when combined with CCTA-derived anatomical or physiological indices (17–19). However, in datasets containing multiple vessels from the same patient, vessel-level allocation or inference may not adequately account for shared patient-specific imaging, acquisition, and biological characteristics. It therefore remains uncertain whether the reported incremental discrimination persists when training and validation cohorts are separated strictly at the patient level and within-patient vessel clustering is explicitly considered. To address this gap, we evaluated lesion-specific PCAT radiomics alone and in combination with DS, CT-FFR, and conventional FAI using patient-level cohort allocation, training-restricted model development, patient-grouped resampling, and cluster-adjusted inference, with invasive FFR as the reference standard.

## 2 Methods

### 2.1 Study design

This retrospective study screened 21,438 consecutive patients with suspected or known stable CAD who underwent clinically indicated CCTA between January 2021 and January 2024. All examinations were performed as part of routine care, and no testing or treatment was assigned by the study protocol. Patients were eligible if they underwent ICA and invasive FFR within 30 days of CCTA, had at least one stenotic native coronary artery evaluated using invasive FFR, and had complete clinical, imaging, and functional assessment data. The exclusion criteria were poor CCTA image quality or failed coronary-tree extraction, unavailable original thin-slice CCTA data, prior target-vessel revascularization without an analyzable native segment, previous coronary stent implantation, CABG, pacemaker or artificial valve implantation, allergy or contraindication to iodinated contrast medium, previous myocardial infarction or unstable angina, severe coronary calcification preventing reliable lumen assessment, complete coronary occlusion on CCTA, or another prespecified condition preventing complete analysis. Patients meeting multiple exclusion criteria were assigned to a single primary exclusion category in the screening audit. Of the 21,438 patients screened, 21,329 did not undergo ICA or invasive FFR, and 10 had an interval of more than 30 days between CCTA and ICA or FFR. The remaining 99 patients underwent detailed eligibility assessment. Under the mutually exclusive primary-reason classification, 18 patients were excluded because of poor CCTA image quality (*n* = 2), unavailable original thin-slice CCTA data (*n* = 15), or prior target-vessel revascularization (*n* = 1). Consequently, 81 patients with 107 target vessels were included in the analysis (Figure 1 and Supplementary Table S1). The study protocol was reviewed and approved by the institutional ethics committee and conducted in accordance with relevant guidelines and the Declaration of Helsinki. All data were anonymized before analysis and handled confidentially to protect participant privacy.

**Figure 1:**
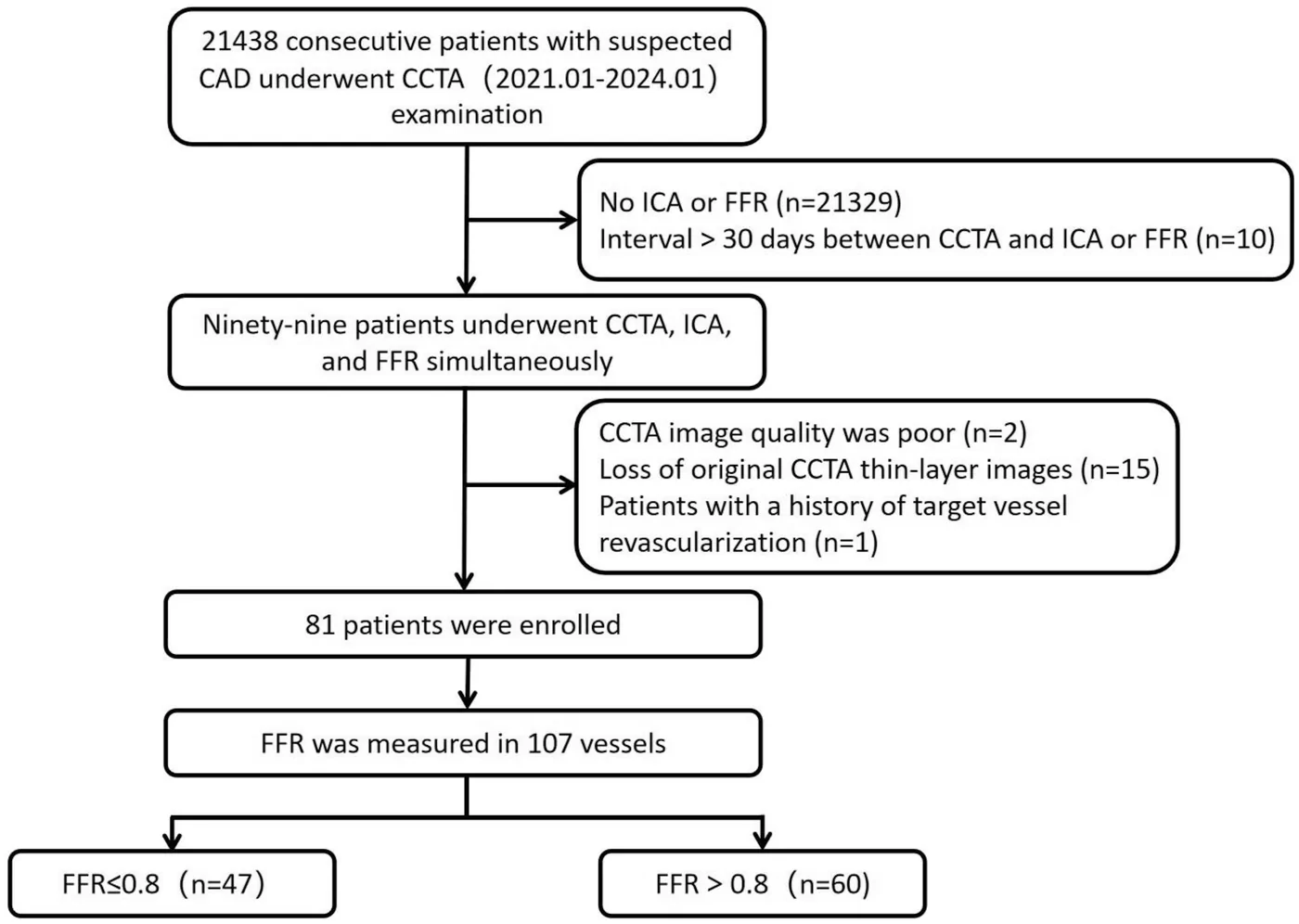
Flowchart of patient selection according to the predefined inclusion and exclusion criteria.

### 2.2 Image acquisition and reconstruction

All CCTA examinations were performed using an Aquilion ONE 320-detector-row CT system (Toshiba/Canon Medical Systems) with prospective or retrospective ECG gating selected according to heart rate and rhythm. Breath-hold training was performed before acquisition. Metoprolol was administered when the pre-scan heart rate exceeded 65 beats/min and no contraindication was present. Sublingual nitroglycerin (0.4 mg) was administered 3–5 min before scanning when clinically appropriate. Iopromide (370 mg iodine/mL) was administered through an 18–20-G catheter in the right antecubital vein. A contrast volume of 40–60 mL was injected at 4.5–5.5 mL/s, followed by a 30 mL saline chaser at the same rate. Bolus tracking was performed using an ROI in the descending aorta, with acquisition initiated 5 s after attenuation reached 180 HU. The scan range extended from 1 cm below the carina to 1 cm above the diaphragm. The acquisition parameters were 100 kVp, 150–200 mA, 320 × 0.5 mm detector collimation, and a 0.275 s rotation time. Images were reconstructed at an automatically selected and visually confirmed motion-free phase, generally at 70%–80% of the R–R interval. Reconstruction used the FC04 medium-smooth cardiac kernel and standard-level AIDR 3D, with a 0.5 mm slice thickness, 0.3 mm increment, 512 × 512 matrix, patient-adjusted field of view of 180–220 mm, and in-plane pixel spacing of 0.35–0.43 mm.

### 2.3 CCTA-Based stenosis analysis and CT-FFR

CCTA images were analyzed using Vitrea/Vital FX version 6.7.2 and Coronary Doc version 5.1.2 (Shukun Technology). The thin-slice contrast-enhanced series with the best diagnostic quality was selected. For each eligible target vessel, the target lesion was defined as the highest-grade stenosis evaluated using invasive FFR. Vessel centerlines, luminal contours, and stenotic segments were automatically extracted and visually reviewed. DS was calculated as: DS (%) = (reference diameter—minimum luminal diameter)/reference diameter × 100. CT-FFR was calculated using a machine-learning reduced-order model trained on synthetic coronary anatomies with computational fluid dynamics-derived reference values. CT-FFR measurements were matched to the target vessels assessed using invasive FFR. DS ≥70% and CT-FFR ≤0.80 were used as the prespecified conventional thresholds for classification analyses.

### 2.4 ICA and invasive FFR measurement

ICA was performed according to routine clinical protocols. After local anesthesia with 1% lidocaine, the right radial artery was punctured and a 6-F vascular sheath was inserted. Heparin (3,000 U) was administered through the sheath, and coronary angiograms were acquired in standard projections and reviewed by experienced operators. Invasive FFR was measured using a 0.014-inch PressureWire X guidewire and QUANTIEN console (Abbott Vascular). The pressure sensor was zeroed outside the body and equalized with the guiding-catheter aortic pressure at the coronary ostium. After intracoronary administration of 200 μg nitroglycerin, the sensor was positioned 2–3 cm distal to the target lesion. Maximal hyperemia was induced by continuous intravenous infusion of adenosine triphosphate (ATP) disodium at 160 μg/kg/min through a peripheral or brachial vein. The infusion was maintained for 2–3 min and continued until a stable hyperemic plateau was achieved. FFR was calculated as mean distal coronary pressure (Pd) divided by mean aortic pressure (Pa), using the stable lowest Pd/Pa value over at least five consecutive cardiac cycles. FFR ≤0.80 was used to define hemodynamically significant stenosis. After measurement, the pressure wire was pulled back to the coronary ostium to assess pressure drift. An absolute Pd/Pa drift ≤0.03 was considered acceptable. Measurements were repeated after re-equalization when drift exceeded 0.03 or the pressure tracing was unstable. Five measurements were repeated, and none were excluded. The ATP dose, administration route, infusion duration, and repeated measurements were verified against catheterization laboratory medication-administration and physiological records. Invasive FFR measurements were performed in accordance with the Chinese expert consensus on coronary FFR measurement (19). Detailed CCTA acquisition, reconstruction, and invasive FFR protocols are provided in Supplementary Table S2.

### 2.5 Quantitative measurement of FAI

FAI was measured using Easy FAI software (Shukun Technology) with deep-learning coronary artery segmentation. PCAT was defined as tissue within a radial distance from the external vessel wall equal to the local vessel diameter and with attenuation values from −190 to −30 HU. FAI was calculated as the mean CT attenuation of all included PCAT voxels. Conventional FAI was measured in fixed proximal coronary segments: 0–40 mm from the vessel origin for the LAD and LCx and 10–50 mm for the RCA. The proximal 10 mm of the RCA was excluded to minimize the influence of the aortic wall. The left main coronary artery was not analyzed because of its variable length. These fixed segments were used only for conventional FAI measurement and were distinct from the lesion-specific PCAT ROIs used for radiomic analysis (20, 21).

### 2.6 PCAT radiomics characterization and model development

Radiomic analysis was performed using lesion-specific three-dimensional PCAT ROIs on the Numericon Research Platform. For each target LAD, LCx, or RCA lesion, the longitudinal ROI extended from the proximal to the distal shoulder of the stenosis, and the radial ROI extended outward from the external vessel wall by a distance equal to the local vessel diameter. Only voxels with attenuation values from −190 to −30 HU were retained, and each ROI was required to contain at least 64 voxels. PCAT masks were generated automatically and reviewed by two cardiovascular radiologists blinded to invasive FFR results. Incorrect vessel-wall delineation or inclusion of nonfat tissue was manually corrected before feature extraction. Segmentation was therefore considered automated with blinded visual quality control rather than fully automated. Images were resampled to isotropic 1.0 × 1.0 × 1.0 mm voxels using B-spline interpolation, whereas masks were resampled using nearest-neighbor interpolation. Attenuation-based resegmentation from −190 to −30 HU was applied after resampling. Gray levels were discretized using a fixed bin width of 25 HU. No intensity normalization was applied because CT attenuation was expressed on an absolute HU scale. Features were extracted from the original images and from eight wavelet decompositions, five Laplacian-of-Gaussian filters (*σ* = 1.0–5.0 mm), mathematical transformations, gradient images, and three-dimensional local binary pattern images. Feature classes included first-order, shape, GLCM, GLDM, GLRLM, GLSZM, and NGTDM features. A total of 1,874 features were extracted per vessel using Python 3.8, PyRadiomics 3.0.1, and SimpleITK 2.2.1. Detailed ROI definitions and preprocessing settings are provided in Supplementary Table S3. During retrospective data analysis, the dataset was randomly split at the patient level into training and validation cohorts in a 7:3 ratio. All vessels from the same patient were assigned to the same cohort. The validation cohort was not used for preprocessing, feature selection, coefficient estimation, hyperparameter tuning, or cutoff selection. Features with more than 10% unavailable values in the training cohort were prespecified for exclusion, and remaining missing values were imputed using the training-set median. Zero-variance features were removed. Radiomic features were standardized using training-set means and SDs, which were subsequently applied unchanged to the validation cohort. Pairwise Pearson correlations were calculated in the training cohort. For each feature pair with |r| ≥0.90, the feature with the larger mean absolute correlation with the remaining features was removed. The retained features were entered into binomial LASSO logistic regression with *α* = 1 and invasive FFR ≤0.80 coded as the positive outcome. A sequence of 100 logarithmically spaced *λ* values from *λ*_max to 0.0001 × *λ*_max was evaluated using patient-grouped 10-fold CV with binomial deviance and a random seed of 20260825. The largest *λ* within one standard error of the minimum cross-validated deviance (*λ*_1SE) was selected. The LASSO linear predictor at *λ*_1SE was retained as the Radscore without post-selection coefficient refitting. The selected features, scaling parameters, coefficients, intercept, and cutoff were applied unchanged to the validation cohort. Seven candidate classifiers were evaluated: LASSO logistic regression, linear SVM, radial-basis-function SVM, random forest, gradient boosting, K-nearest neighbors, and decision tree. Classifiers were compared using patient-grouped nested 5 × 5-fold CV repeated 20 times, with identical outer resampling indices. Preprocessing, feature selection, hyperparameter tuning, and cutoff selection were repeated within each outer training fold, and performance was evaluated using out-of-fold predictions. The classifier with the highest mean cross-validated AUC was selected, with model parsimony considered when performance estimates were similar. After classifier selection, the final Radscore model was fitted in the complete training cohort using the separate patient-grouped 10-fold CV procedure described above. Seven diagnostic models were evaluated: DS, CT-FFR, FAI, Radscore, DS+ Radscore, CT-FFR+ Radscore, and FAI+ Radscore. The combined models were fitted using logistic regression in the training cohort and applied to the validation cohort without refitting. DS ≥70% and CT-FFR ≤0.80 were prespecified conventional thresholds. Cutoffs for FAI, Radscore, and the combined models were determined in the training cohort and applied unchanged to the validation cohort. For the sensitivity analysis, lesions were classified as completely within, partly within, or outside the fixed proximal FAI region according to their proximal and distal shoulders. The analysis was repeated in lesions located completely within that region using the original fitted models and training-derived cutoffs without refitting.

### 2.7 Statistical analysis

Continuous variables were summarized as mean ± SD or median (IQR), as appropriate, and categorical variables as *n* (%). Patient-level characteristics were compared between patients with at least one FFR-positive vessel and those with exclusively FFR-negative vessels. Continuous variables were compared using independent-samples *t*-tests, whereas categorical variables were compared using Pearson *χ*^2^ tests or Fisher's exact tests. Vessel-level analyses accounted for the clustering of multiple vessels within patients. Continuous variables presented as mean ± SD were analyzed using linear mixed-effects models with a patient-specific random intercept. Binary and categorical variables were analyzed using GEE. Skewed continuous variables were summarized as median (IQR), with cluster-adjusted median differences reported. Effect estimates were presented as mean differences, median differences, or ORs with 95% CIs, as appropriate. Model discrimination was evaluated using the AUC. Threshold-dependent performance was assessed using accuracy, sensitivity, specificity, PPV, NPV, TP, FN, FP, and TN. The 95% CIs for AUCs and classification measures were estimated using 2,000 patient-cluster bootstrap replicates. Patients were resampled as clusters, with all vessels from each sampled patient retained together. Differences in AUC between each combined model and its corresponding conventional model were evaluated using paired patient-cluster bootstrap distributions. The same approach was used for the analysis restricted to lesions located completely within the fixed proximal FAI region. All analyses were performed using R version 4.3.3. The principal packages were glmnet 4.1–8 for LASSO regression, pROC 1.18.5 for ROC analysis, geepack 1.3–12 for GEE, lme4 1.1–35.3 for mixed-effects models, and rsample 1.2.1 and tidymodels 1.2.0 for resampling and model evaluation. All tests were two-sided, and *P* < 0.05 was considered statistically significant.

## 3 Results

### 3.1 Baseline characteristics

Among 21,438 patients who underwent CCTA during the study period, 21,329 did not undergo ICA or invasive FFR, and 10 had an interval of more than 30 days between CCTA and ICA or FFR. Of the remaining 99 patients, 18 were excluded during detailed eligibility assessment, leaving 81 patients with 107 target vessels for analysis (Figure 1 and Supplementary Table S1). The included patients had a mean age of 61.75 ± 9.15 years, and 52 (64.2%) were male. Hypertension was present in 47 patients (58.0%), diabetes in 27 (33.3%), hyperlipidemia in 20 (24.7%), and current smoking in 16 (19.8%). Forty patients had at least one target vessel with FFR ≤0.80, whereas all target vessels had FFR >0.80 in the remaining 41 patients. Current smoking was more frequent among patients with at least one FFR-positive vessel than among those with exclusively FFR-negative vessels (30.0% vs. 9.8%, *P* = 0.022). No statistically significant between-group differences were observed in age, sex, BMI, diabetes, hypertension, hyperlipidemia, or family history of CAD (all *P* ≥ 0.132; Table 1).

**Table 1:** Baseline characteristics of patients.

Characteristic	Overall (*n* = 81)	Patients with ≥1 target vessel FFR ≤0.80 (*n* = 40)	Patients with all target vessels FFR >0.80 (*n* = 41)	Statistic	*P* value
Age, years	61.75 ± 9.15	60.20 ± 9.73	63.27 ± 8.40	*t* = −1.520	0.132
Male, *n* (%)	52 (64.2)	28 (70.0)	24 (58.5)	*χ*^2^ = 1.158	0.282
Body mass index, kg/m^2^	24.58 ± 2.92	24.61 ± 2.74	24.54 ± 3.13	*t* = 0.103	0.918
Diabetes, *n* (%)	27 (33.3)	11 (27.5)	16 (39.0)	χ^2^ = 1.210	0.271
Hypertension, *n* (%)	47 (58.0)	21 (52.5)	26 (63.4)	χ^2^ = 0.990	0.320
Hyperlipidemia, *n* (%)	20 (24.7)	9 (22.5)	11 (26.8)	χ^2^ = 0.204	0.651
Current smoker, *n* (%)	16 (19.8)	12 (30.0)	4 (9.8)	χ^2^ = 5.235	0.022
Family history of CAD, *n* (%)	3 (3.7)	1 (2.5)	2 (4.9)	Fisher's exact test	1.000

CAD, coronary artery disease; FFR, fractional flow reserve.

### 3.2 Cohort allocation and vessel-level characteristics

The patient-level 7:3 split yielded a training cohort of 57 patients with 75 target vessels and a validation cohort of 24 patients with 32 target vessels, including 33 and 14 FFR-positive vessels, respectively. No patient contributed vessels to both cohorts. Of the 81 patients, 59 contributed one vessel, 18 contributed two vessels, and four contributed three vessels (Supplementary Table S4).

After accounting for within-patient clustering, FFR-positive vessels had greater DS (75.9% ± 11.1% vs. 64.0% ± 11.4%; mean difference, 11.9 percentage points; 95% CI, 7.4–16.4; *P* < 0.001) and lower CT-FFR values (0.72 ± 0.09 vs. 0.85 ± 0.09; mean difference, −0.13; 95% CI, −0.17 to −0.09; *P* < 0.001) than FFR-negative vessels. DS ≥70% was observed in 72.3% and 40.0% of FFR-positive and FFR-negative vessels, respectively (OR, 3.76; 95% CI, 1.55–9.13; *P* = 0.003). FAI values were less negative in FFR-positive vessels (median, −78 HU; IQR, −87 to −69 HU) than in FFR-negative vessels (median, −85.5 HU; IQR, −92.75 to −76.25 HU), with a cluster-adjusted median difference of 7.4 HU (95% CI, 1.6–13.2 HU; *P* = 0.013). PCAT volume and lesion distribution did not differ significantly by FFR status. The two positively weighted radiomic features were higher in FFR-positive vessels, whereas the negatively weighted feature was lower (Table 2).

**Table 2:** Vessel-level characteristics according to invasive FFR status.

Variable	Overall (*n* = 107)	FFR >0.80 (*n* = 60)	FFR ≤0.80 (*n* = 47)	Cluster-adjusted effect estimate (95% CI)	Cluster-adjusted *P* value
Invasive FFR	0.81 ± 0.11	0.89 ± 0.05	0.70 ± 0.08	Mean difference, −0.19 (−0.22 to −0.16)	<0.001
DS, %	69.2 ± 12.7	64.0 ± 11.4	75.9 ± 11.1	Mean difference, 11.9 (7.4 to 16.4)	<0.001
DS ≥70%, *n* (%)	58 (54.2)	24 (40.0)	34 (72.3)	OR, 3.76 (1.55 to 9.13)	0.003
CT-FFR	0.79 ± 0.11	0.85 ± 0.09	0.72 ± 0.09	Mean difference, −0.13 (−0.17 to −0.09)	<0.001
Lesion location, *n* (%)					0.193
LAD	71 (66.4)	39 (65.0)	32 (68.1)	Reference	—
LCx	13 (12.1)	5 (8.3)	8 (17.0)	OR, 2.05 (0.58 to 7.21)	0.265
RCA	23 (21.5)	16 (26.7)	7 (14.9)	OR, 0.50 (0.17 to 1.48)	0.211
PCAT volume, mm³	1,968.01 ± 547.72	1,997.28 ± 520.15	1,930.64 ± 584.59	Mean difference, −66.6 (−284.0 to 150.7)	0.547
FAI, HU	−84 (−92 to −75)	−85.5 (−92.75 to −76.25)	−78 (−87 to −69)	Median difference, 7.4 HU (1.6 to 13.2)	0.013
exponential_firstorder_Minimum, z score	−0.059 (−0.992 to 0.507)	−0.249 (−1.193 to 0.085)	0.481 (−0.171 to 0.804)	Median difference, 0.73 (0.36 to 1.10)	<0.001
original_gldm_SmallDependenceLowGrayLevelEmphasis, z score	−0.096 (−0.971 to 0.533)	−0.490 (−1.070 to 0.293)	0.111 (−0.790 to 0.758)	Median difference, 0.60 (0.11 to 1.09)	0.017
wavelet-HHH_glszm_GrayLevelNonUniformity, z score	−0.127 ± 0.993	0.075 ± 1.088	−0.385 ± 0.796	Mean difference, −0.46 (−0.84 to −0.08)	0.018

CI, confidence interval; CT-FFR, computed tomography-derived fractional flow reserve; DS, diameter stenosis; FAI, fat attenuation index; FFR, fractional flow reserve; GLDM, gray-level dependence matrix; GLSZM, gray-level size zone matrix; HU, Hounsfield unit; LAD, left anterior descending artery; LCx, left circumflex artery; OR, odds ratio; PCAT, pericoronary adipose tissue; RCA, right coronary artery.

### 3.3 Radiomic feature selection and radscore development

Among the 1,874 extracted radiomic features, none were excluded because of nonfinite values or zero variance. Correlation filtering performed exclusively in the training cohort retained 450 features. Patient-grouped 10-fold CV yielded *λ*_min = 0.019 and *λ*_1SE = 0.052, with three features retaining nonzero coefficients under the one-standard-error rule. The resulting score was: Radscore = −0.238 + 0.421Z_1_ + 0.683Z_2_−0.512Z₃, where Z_1_, Z_2_, and Z_3_ were the training-standardized values of original_gldm_SmallDependenceLowGrayLevelEmphasis, exponential_firstorder_Minimum, and wavelet-HHH_glszm_GrayLevelNonUniformity, respectively. The selected features, scaling parameters, coefficients, intercept, and cutoff were applied unchanged to the validation cohort (Supplementary Table S5).

Among the seven classifiers evaluated using patient-grouped nested CV, LASSO logistic regression had the highest mean out-of-fold AUC (0.791; 95% cluster-bootstrap CI, 0.671–0.879), with an accuracy of 0.773 (95% CI, 0.666–0.854). The AUCs of the other classifiers ranged from 0.668 for the decision tree to 0.782 for the linear SVM. LASSO logistic regression was selected for Radscore development based on its cross-validated AUC and model parsimony (Supplementary Table S6).

### 3.4 Diagnostic performance in the training and validation cohorts

Model discrimination and threshold-dependent performance are summarized in Table 3. In the training cohort, the AUCs were 0.678 for DS, 0.781 for CT-FFR, 0.643 for FAI, and 0.789 for Radscore. The DS+ Radscore, CT-FFR+ Radscore, and FAI+ Radscore models had AUCs of 0.844, 0.854, and 0.811, respectively. Compared with the corresponding conventional models, the cluster-bootstrap *Δ*AUCs were 0.166 (95% CI, 0.052–0.276; *P* = 0.004), 0.073 (95% CI, 0.008–0.139; *P* = 0.028), and 0.168 (95% CI, 0.017–0.319; *P* = 0.030), respectively.

**Table 3:** Diagnostic performance of models for identifying hemodynamically significant stenosis.

Panel A. Model discrimination				
Cohort	Model	AUC (95% patient-cluster bootstrap CI)	*Δ*AUC versus reference model (95% CI)	*P* value
Training: 57 patients, 75 vessels, 33 FFR-positive vessels	DS	0.678 (0.550–0.798)	Reference	—
Training	CT-FFR	0.781 (0.668–0.881)	Reference	—
Training	FAI	0.643 (0.501–0.773)	Reference	—
Training	Radscore	0.789 (0.667–0.890)	—	—
Training	DS + Radscore	0.844 (0.742–0.924)	0.166 (0.052–0.276)	0.004
Training	CT-FFR + Radscore	0.854 (0.754–0.930)	0.073 (0.008–0.139)	0.028
Training	FAI + Radscore	0.811 (0.692–0.905)	0.168 (0.017–0.319)	0.03
Validation: 24 patients, 32 vessels, 14 FFR-positive vessels	DS	0.619 (0.412–0.801)	Reference	—
Validation	CT-FFR	0.762 (0.561–0.914)	Reference	—
Validation	FAI	0.585 (0.347–0.797)	Reference	—
Validation	Radscore	0.684 (0.451–0.873)	—	—
Validation	DS + Radscore	0.812 (0.609–0.944)	0.193 (0.018–0.367)	0.03
Validation	CT-FFR + Radscore	0.879 (0.694–0.981)	0.117 (0.005–0.229)	0.041
Validation	FAI + Radscore	0.710 (0.478–0.886)	0.125 (−0.083 to 0.333)	0.238

Panel B. Threshold-dependent diagnostic performance							
Cohort	Model	Cutoff applied	Accuracy, % (95% cluster-bootstrap CI)	Sensitivity, % (95% cluster-bootstrap CI)	Specificity, % (95% cluster-bootstrap CI)	PPV, % (95% cluster-bootstrap CI)	NPV, % (95% cluster-bootstrap CI)
Training	DS	≥70%	66.7 (55.3–77.6)	72.7 (54.8–87.5)	61.9 (47.6–75.9)	60.0 (43.3–75.4)	74.3 (60.4–87.5)
Training	CT-FFR	≤0.80	77.3 (68.1–86.1)	87.9 (75.0–97.3)	69.0 (55.8–82.1)	69.0 (55.3–81.8)	87.9 (76.7–97.1)
Training	FAI	≥−79 HU	64.0 (52.2–75.0)	60.6 (40.6–77.8)	66.7 (52.3–80.4)	58.8 (40.0–75.6)	68.3 (54.3–81.4)
Training	Radscore	≥0.59	77.3 (67.6–86.3)	63.6 (44.4–80.5)	88.1 (77.1–97.4)	80.8 (62.5–95.2)	75.5 (64.8–85.7)
Training	DS + Radscore	≥0.45	78.7 (68.5–87.7)	81.8 (66.7–94.1)	76.2 (63.0–88.1)	73.0 (57.6–86.5)	84.2 (71.8–94.7)
Training	CT-FFR + Radscore	≥0.59	84.0 (75.3–91.7)	78.8 (62.9–92.1)	88.1 (77.3–97.4)	83.9 (68.8–96.0)	84.1 (74.4–93.8)
Training	FAI + Radscore	≥0.48	78.7 (67.7–88.1)	75.8 (58.6–89.7)	81.0 (68.2–92.2)	75.8 (59.1–89.7)	81.0 (68.4–91.9)
Validation	DS	≥70%	62.5 (44.4–80.0)	71.4 (46.7–92.9)	55.6 (33.3–77.8)	55.6 (33.3–76.5)	71.4 (46.2–93.3)
Validation	CT-FFR	≤0.80	75.0 (58.1–90.3)	78.6 (57.1–100.0)	72.2 (50.0–91.7)	68.8 (46.2–90.0)	81.3 (60.0–100.0)
Validation	FAI	≥−79 HU	56.3 (40.6–72.7)	50.0 (23.8–77.8)	61.1 (37.5–83.3)	50.0 (26.7–72.7)	61.1 (40.0–84.2)
Validation	Radscore	≥0.59	68.8 (51.5–85.7)	57.1 (26.7–85.7)	77.8 (57.1–94.7)	66.7 (37.5–91.7)	70.0 (50.0–90.0)
Validation	DS + Radscore	≥0.45	71.9 (52.8–89.7)	71.4 (46.2–92.9)	72.2 (50.0–92.9)	66.7 (40.0–90.0)	76.5 (53.8–94.4)
Validation	CT-FFR + Radscore	≥0.59	71.9 (53.3–88.9)	50.0 (20.0–80.0)	88.9 (72.2–100.0)	77.8 (42.9–100.0)	69.6 (50.0–89.5)
Validation	FAI + Radscore	≥0.48	65.6 (45.7–84.4)	57.1 (26.7–86.7)	72.2 (50.0–92.9)	61.5 (33.3–86.7)	68.4 (45.0–90.0)

AUC, area under the receiver operating characteristic curve; CI, confidence interval; CT-FFR, computed tomography-derived fractional flow reserve; DS, diameter stenosis; FAI, fat attenuation index; FFR, fractional flow reserve; HU, Hounsfield unit; NPV, negative predictive value; PPV, positive predictive value; Radscore, radiomics score.

Symbol: *Δ*AUC, difference in AUC relative to the specified reference model.

In the validation cohort, Radscore alone had an AUC of 0.684 (95% CI, 0.451–0.873), with the CI including 0.50. The CT-FFR+ Radscore model had the largest estimated AUC among the evaluated models (0.879; 95% CI, 0.694–0.981), compared with 0.762 (95% CI, 0.561–0.914) for CT-FFR alone (*Δ*AUC, 0.117; 95% CI, 0.005–0.229; *P* = 0.041). The DS+ Radscore model also had a higher AUC than DS alone (0.812 vs. 0.619; *Δ*AUC, 0.193; 95% CI, 0.018–0.367; *P* = 0.030). For FAI+ Radscore vs. FAI alone, the *Δ*AUC was 0.125 (95% CI, −0.083 to 0.333; *P* = 0.238).

At the training-derived cutoffs, CT-FFR+ Radscore had lower validation accuracy (71.9% vs. 75.0%), sensitivity (50.0% vs. 78.6%), and NPV (69.6% vs. 81.3%) than CT-FFR alone, but higher specificity (88.9% vs. 72.2%) and PPV (77.8% vs. 68.8%). The CT-FFR+ Radscore model classified seven vessels as TP, seven as FN, two as FP, and 16 as TN, compared with 11 TP, three FN, five FP, and 13 TN for CT-FFR alone (Supplementary Table S7).

### 3.5 Analysis restricted to lesions within the fixed proximal FAI region

Lesion–ROI concordance and the ROI-restricted analysis are summarized in Supplementary Table S8. Of the 107 target lesions, 83 (77.6%) were located completely within the fixed proximal FAI region, 11 (10.3%) were partly within the region, and 13 (12.1%) were outside it. The restricted analysis included 58 training vessels, of which 27 were FFR-positive, and 25 validation vessels, of which 11 were FFR-positive.

In the restricted training subset, CT-FFR+ Radscore had an AUC of 0.881 (95% patient-cluster bootstrap CI, 0.786–0.944), sensitivity of 85.2%, and specificity of 90.3%. In the restricted validation subset, the corresponding estimates were 0.897 (95% CI, 0.717–0.978), 63.6% (95% CI, 31.6%–86.8%), and 92.9% (95% CI, 64.2%–99.2%), respectively. CT-FFR alone had a validation AUC of 0.781, sensitivity of 81.8%, and specificity of 71.4% in this subset. The validation estimates remained imprecise, particularly for sensitivity, as indicated by the wide CIs.

## 4 Discussion

In this retrospective study, lesion-specific three-dimensional PCAT radiomics provided information that was complementary to established CCTA-derived anatomical and functional indices for identifying FFR-defined hemodynamically significant coronary stenosis. The principal novelty lies in analyzing PCAT immediately surrounding each target stenosis rather than relying exclusively on fixed proximal coronary segments, thereby improving spatial correspondence between the tissue phenotype and the lesion-specific physiological reference (22, 23). The study also evaluated the Radscore alongside DS, CT-FFR, and conventional FAI and applied patient-level cohort allocation, training-restricted feature selection, and cluster-adjusted inference to account for patients contributing multiple vessels. The higher validation AUCs observed after combining Radscore with CT-FFR or DS suggest that perilesional adipose-tissue texture may provide information not fully represented by luminal narrowing or computational estimates of coronary physiology (24, 25). Clinically, radiomic features can be derived from routinely acquired CCTA images without additional scanning, contrast administration, or radiation exposure (26, 27). If confirmed in independent cohorts, lesion-specific PCAT radiomics could therefore serve as an adjunctive CCTA marker for refining lesion-level risk stratification and identifying stenoses that warrant further functional assessment. However, the present findings support a complementary role rather than replacement of CT-FFR or invasive FFR, because the clinical interpretation of a combined model depends not only on discrimination but also on its sensitivity, specificity, and intended position within the diagnostic pathway.

At the vessel level, FFR-positive stenoses had greater DS and lower CT-FFR than FFR-negative stenoses, with adjusted mean differences of 11.9 percentage points and −0.13, respectively. The direction and magnitude of these differences are consistent with the established association between anatomical stenosis severity, pressure loss, and lesion-specific physiological significance (28, 29). FAI was also less negative in FFR-positive vessels, with a cluster-adjusted median difference of 7.4 HU, whereas PCAT volume did not differ significantly between the two groups. This contrasting pattern indicates that attenuation characteristics and internal tissue composition may be more closely associated with FFR status than the overall amount of adipose tissue surrounding the coronary artery. Previous CCTA studies have interpreted less-negative FAI as an imaging correlate of altered lipid composition, increased aqueous content, and inflammatory remodeling within perivascular adipose tissue (30, 31). The present findings are compatible with this interpretation but establish only an imaging association and do not demonstrate an inflammatory or causal mechanism. The selected radiomic features provided additional characterization of PCAT beyond its mean attenuation. Original_gldm_SmallDependenceLowGrayLevelEmphasis and exponential_firstorder_Minimum had positive coefficients and were higher in FFR-positive vessels, whereas wavelet-HHH_glszm_GrayLevelNonUniformity had a negative coefficient and was lower in these vessels. These features quantify different aspects of low-gray-level dependence, transformed intensity distribution, and spatial variation within high-frequency wavelet components (32). Their combined contribution suggests that FFR-positive and FFR-negative lesions may differ not only in average PCAT attenuation but also in the spatial organization and heterogeneity of voxel intensities. Such information is not captured by conventional FAI, which reduces the entire measurement region to a single mean attenuation value. Nevertheless, the biological meanings of individual radiomic features should be interpreted cautiously because their values depend on image transformation, resampling, discretization, and mathematical feature definitions (33). They are therefore best regarded as reproducible quantitative imaging descriptors rather than direct measures of adipocyte morphology, tissue inflammation, or histopathological composition.

The model comparisons highlight an important distinction between discrimination across the full range of predicted values and classification at a specific cutoff (34). In the validation cohort, Radscore alone had an AUC of 0.684, with a 95% CI of 0.451 to 0.873 that included 0.50. Its standalone discrimination was therefore uncertain. When Radscore was combined with CT-FFR, the AUC increased from 0.762 to 0.879, with a cluster-bootstrap *Δ*AUC of 0.117. Combining Radscore with DS produced a similar pattern, with the AUC increasing from 0.619 to 0.812 and a *Δ*AUC of 0.193. In contrast, FAI + Radscore did not have a statistically significantly higher AUC than FAI alone. This differential pattern may reflect the extent of information shared by the component predictors. FAI and Radscore are both derived from PCAT, whereas DS and CT-FFR describe anatomical narrowing and estimated lesion physiology, respectively. Radiomic texture may consequently provide more nonredundant information when combined with DS or CT-FFR than when combined with mean PCAT attenuation. The threshold-dependent findings provide a more nuanced interpretation. At the training-derived cutoff, CT-FFR + Radscore had higher validation specificity than CT-FFR alone (88.9% vs. 72.2%) and a higher PPV (77.8% vs. 68.8%). However, its accuracy was lower (71.9% vs. 75.0%), as were its sensitivity (50.0% vs. 78.6%) and NPV (69.6% vs. 81.3%). The higher AUC therefore reflected improved ranking over the full prediction range rather than uniformly better classification at the selected cutoff. The analysis restricted to lesions completely within the fixed proximal FAI region showed a comparable specificity-oriented pattern, with an AUC of 0.897, sensitivity of 63.6%, and specificity of 92.9% for CT-FFR + Radscore. These findings indicate that the appropriate operating threshold would depend on the intended clinical task. A rule-out pathway would require greater emphasis on sensitivity and NPV, whereas a confirmatory strategy could reasonably prioritize specificity and PPV to reduce false-positive referrals for invasive assessment (35). Accordingly, the Radscore should be interpreted as an adjunctive marker for refining lesion-level assessment when used with established CCTA metrics, particularly CT-FFR or DS, rather than as a standalone diagnostic marker. Its potential clinical role requires confirmation in larger external cohorts.

These findings can be interpreted in the context of recent studies examining PCAT radiomics as a marker of lesion-specific ischemia. Wen et al. reported that combining lesion-based PCAT radiomics with CCTA was associated with a higher AUC for identifying FFR-positive stenosis than CCTA alone (0.812 vs. 0.599) (36). Consistent with this pattern, DS+ Radscore in our validation cohort had an AUC of 0.812, compared with 0.619 for DS alone. Whereas Wen et al. used lesion-level allocation and manual PCAT segmentation, our analysis used patient-level cohort separation, automated segmentation with blinded quality control, training-restricted LASSO selection, and cluster-adjusted inference. In a subsequent study, Wen et al. found comparable performance between fixed proximal vessel-based and lesion-based PCAT segmentations (23). Accordingly, our findings should not be interpreted as evidence that lesion-specific segmentation is intrinsically superior. Rather, our lesion-to-ROI concordance analysis showed that applying the unchanged models to lesions completely within the fixed FAI region produced results directionally consistent with the primary analysis. Feng et al. reported AUCs of approximately 0.82 for a PCAT radiomics signature and 0.83 in an invasive-FFR subgroup, with better discrimination than conventional FAI (37). Although our Radscore had a lower validation AUC of 0.684, CT-FFR+ Radscore achieved an AUC of 0.879, suggesting that the observed value of radiomics was primarily complementary rather than standalone. This result closely parallels that of Yu et al., who reported validation AUCs of 0.875 for CT-FFR combined with PCAT radiomics and 0.791 for CT-FFR alone (24). Zhou et al. similarly reported testing AUCs of 0.696, 0.740, and 0.809 for conventional, radiomics, and combined models, respectively (38). Our DS+ Radscore result was directionally consistent, although the larger multicenter design of Zhou et al. provided broader evidence. Collectively, these studies and our findings support an association between PCAT texture and FFR-defined ischemia, while our patient-grouped analysis additionally demonstrates that higher discrimination may coexist with lower sensitivity and NPV at a training-derived cutoff. This distinction is important when considering how such models might be positioned within future diagnostic pathways.

This study has several limitations. First, its single-center retrospective design and restriction to patients who underwent CCTA, ICA, and invasive FFR within 30 days may have introduced selection and spectrum bias, limiting generalizability. Second, the validation cohort included only 24 patients, 32 vessels, and 14 FFR-positive vessels. Several estimates therefore had wide CIs, the CI for Radscore alone included 0.50, and subgroup analyses, including evaluation of the CT-FFR gray zone, were not feasible. Moreover, validation was internal, with no independent external cohort. Third, the higher AUC of CT-FFR+ Radscore did not translate into uniformly better classification; at the training-derived cutoff, sensitivity and NPV were lower than those of CT-FFR alone. The findings therefore indicate higher discrimination rather than clinical superiority. Fourth, all CCTA examinations used a single scanner and reconstruction protocol, and cross-vendor reproducibility remains unknown. Medication use and longitudinal risk-factor control were not comprehensively characterized. Fifth, although patient-level allocation, grouped resampling, and cluster-adjusted inference accounted for multiple vessels per patient, performance was evaluated at the vessel level and may not directly reflect patient-level decision-making. Finally, the selected radiomic features lacked histopathological validation, and formal interobserver segmentation repeatability was not assessed. Larger prospective multicenter studies should externally validate the frozen models and thresholds across imaging platforms, assess calibration and clinical utility, and evaluate parsimonious integrated models before clinical implementation.

## 5 Conclusion

In this retrospective cohort, selected lesion-specific PCAT radiomic features differed between FFR-positive and FFR-negative vessels. Adding Radscore to CT-FFR or DS was associated with higher discrimination in the held-out internal validation cohort, whereas no statistically significant improvement in discrimination was observed for FAI+ Radscore. Threshold-dependent classification performance did not improve uniformly, and CT-FFR+ Radscore showed lower sensitivity and negative predictive value than CT-FFR alone at the training-derived cutoffs. These exploratory findings warrant validation in larger independent cohorts before clinical application.

## Data Availability

The raw data supporting the conclusions of this article will be made available by the authors, without undue reservation.
