# Supplementary material for: Incremental diagnostic value of coronary CT angiography-derived pericoronary adipose tissue radiomics for identifying hemodynamically significant coronary stenosis
Source: Front Cardiovasc Med. 2026 Sep 29;13:1943961. doi: 10.3389/fcvm.2026.1943961 (PMC13657987; doi:10.3389/fcvm.2026.1943961)
Supplement: Supplementary file 1 [file Datasheet1.docx]

Supplementary Table S1. Patient screening and mutually exclusive exclusion reasons

| Screening stage or primary exclusion reason | Number, n |
| --- | --- |
| Patients with suspected or known stable CAD who underwent CCTA during the study period | 21,438 |
| Excluded before detailed eligibility assessment | 21,339 |
| No ICA or invasive FFR | 21,329 |
| Interval >30 days between CCTA and ICA or invasive FFR | 10 |
| Patients undergoing detailed eligibility assessment | 99 |
| Excluded after detailed eligibility assessment | 18 |
| Poor CCTA image quality or failed coronary-tree extraction | 2 |
| Original thin-slice CCTA data unavailable | 15 |
| Prior target-vessel revascularization without an analyzable native segment | 1 |
| Previous coronary stent implantation | 0 |
| Previous CABG | 0 |
| Pacemaker implantation | 0 |
| Artificial valve implantation | 0 |
| Allergy or contraindication to iodinated contrast medium | 0 |
| Previous myocardial infarction or unstable angina | 0 |
| Severe coronary calcification preventing reliable lumen assessment | 0 |
| Complete coronary occlusion on CCTA | 0 |
| Other prespecified condition preventing complete analysis | 0 |
| Patients included in the final analysis | 81 |
| Target vessels analyzed | 107 |

Abbreviations: CABG, coronary artery bypass grafting; CAD, coronary artery disease; CCTA, coronary computed tomography angiography; FFR, fractional flow reserve; ICA, invasive coronary angiography.

Supplementary Table S2. Imaging acquisition and invasive FFR protocol

Panel A. CCTA acquisition and reconstruction

| Parameter | Overall protocol/value | Training cohort | Validation cohort |
| --- | --- | --- | --- |
| Scanner | Aquilion ONE, 320-detector-row CT (Toshiba/Canon Medical Systems) | Same scanner | Same scanner |
| Postprocessing software | Vitrea/Vital FX 6.7.2; Coronary Doc 5.1.2; Easy FAI | Same | Same |
| ECG gating | Prospective: 66/81 (81.5%); retrospective: 15/81 (18.5%) | 46/57; 11/57 | 20/24; 4/24 |
| Tube voltage | 100 kVp | 100 kVp | 100 kVp |
| Tube current | 150–200 mA | 150–200 mA | 150–200 mA |
| Detector collimation | 320 × 0.5 mm | Same | Same |
| Rotation time | 0.275 s | Same | Same |
| Heart rate during acquisition | 62.1 ± 7.6 beats/min | 61.8 ± 7.4 | 62.8 ± 8.0 |
| β-blocker preparation | Metoprolol when pre-scan heart rate >65 beats/min and no contraindication; 34/81 (42.0%) | 24/57 (42.1%) | 10/24 (41.7%) |
| Sublingual nitroglycerin | 0.4 mg, 3–5 min before acquisition; 77/81 (95.1%) | 54/57 (94.7%) | 23/24 (95.8%) |
| Contrast agent | Iopromide, 370 mg iodine/mL | Same | Same |
| Contrast volume | 40–60 mL | Same | Same |
| Injection rate | 4.5–5.5 mL/s | Same | Same |
| Saline chaser | 30 mL at the same injection rate | Same | Same |
| Venous access | Right antecubital vein, 18–20 G | Same | Same |
| Bolus tracking | Descending aorta ROI; 180-HU trigger; 5-s delay | Same | Same |
| Scan range | 1 cm below the carina to 1 cm above the diaphragm | Same | Same |
| Reconstructed cardiac phase | Motion-free phase selected automatically and visually confirmed; generally 70%–80% of R–R interval | Same | Same |
| Reconstruction kernel | Medium-smooth cardiac kernel, FC04 | Same | Same |
| Iterative reconstruction | AIDR 3D, standard level | Same | Same |
| Slice thickness | 0.5 mm | Same | Same |
| Reconstruction increment | 0.3 mm | Same | Same |
| Matrix | 512 × 512 | Same | Same |
| Reconstruction field of view | Patient-adjusted, 180–220 mm | Same | Same |
| In-plane pixel spacing | 0.35–0.43 mm | Same | Same |
| Missing acquisition/reconstruction metadata | 0/81 (0%) | 0/57 (0%) | 0/24 (0%) |

Panel B. Invasive FFR protocol

| Parameter | Protocol/value | Vessels, n |
| --- | --- | --- |
| Pressure-wire system | PressureWire X 0.014-inch pressure-sensor guidewire with QUANTIEN console (Abbott Vascular) | 107 |
| Zeroing | Pressure-wire transducer zeroed outside the body before insertion | 107 |
| Equalization | Wire pressure equalized with guiding-catheter aortic pressure at the coronary ostium | 107 |
| Wire position | Pressure sensor positioned at least 2–3 cm distal to the target lesion | 107 |
| Intracoronary nitroglycerin | 200 μg administered before pressure recording | 107 |
| Hyperemic agent | Intravenous adenosine triphosphate disodium | 107 |
| ATP dose | 160 μg/kg/min | 107 |
| Administration route | Continuous peripheral or brachial intravenous infusion | 107 |
| Infusion duration | 2–3 min and continued until a stable hyperemic plateau was achieved | 107 |
| Stable hyperemia criterion | Stable lowest Pd/Pa value over ≥5 consecutive cardiac cycles after hemodynamic stabilization | 107 |
| FFR calculation | Mean distal coronary pressure divided by mean aortic pressure during maximal hyperemia | 107 |
| Positivity threshold | FFR ≤0.80 | 107 |
| Pullback assessment | Pressure wire pulled back to the coronary ostium after FFR measurement | 107 |
| Acceptable pressure drift | Absolute Pd/Pa drift ≤0.03 | 107 |
| Repeated measurements | FFR measurement repeated after re-equalization when pressure drift exceeded 0.03 or the pressure tracing was unstable | 5 |
| Excluded FFR measurements | None | 0 |

Abbreviations: AIDR 3D, adaptive iterative dose reduction three-dimensional; ATP, adenosine triphosphate; CCTA, coronary computed tomography angiography; CT, computed tomography; ECG, electrocardiography; FAI, fat attenuation index; FFR, fractional flow reserve; HU, Hounsfield unit; ROI, region of interest.

Pressure symbols: Pa, aortic pressure; Pd, distal coronary pressure.

Supplementary Table S3. PCAT radiomics ROI definition and preprocessing

| Processing component | Required setting |
| --- | --- |
| Source CCTA series | Thin-slice, contrast-enhanced CCTA series with the best diagnostic image quality; 0.5-mm thickness and 0.3-mm increment |
| Radiomics ROI type | Lesion-specific three-dimensional PCAT volume |
| Target lesion | Highest-grade stenosis evaluated by invasive FFR in each target vessel |
| LAD longitudinal boundary | From the proximal shoulder to the distal shoulder of the target LAD lesion |
| LCx longitudinal boundary | From the proximal shoulder to the distal shoulder of the target LCx lesion |
| RCA longitudinal boundary | From the proximal shoulder to the distal shoulder of the target RCA lesion |
| Radial boundary | Tissue extending outward from the external vessel wall by a distance equal to the local vessel diameter |
| PCAT attenuation range | −190 to −30 HU |
| Extraction dimensionality | Three-dimensional |
| Image resampling | Isotropic 1.0 × 1.0 × 1.0 mm voxels |
| Image interpolation | B-spline interpolation |
| Mask interpolation | Nearest-neighbor interpolation |
| Resegmentation | −190 to −30 HU after resampling |
| Minimum ROI size | ≥64 included voxels |
| Gray-level discretization | Fixed bin width |
| Bin width | 25 HU |
| Approximate number of bins | 7–8 across the specified fat attenuation range |
| Image intensity normalization | None before feature extraction because CT attenuation is expressed on an absolute HU scale |
| Feature standardization | Z-score standardization using training-set mean and SD only; the same parameters were applied unchanged to validation data |
| Original image | Included |
| Wavelet filtering | Eight decompositions: LLL, LLH, LHL, LHH, HLL, HLH, HHL, and HHH |
| Laplacian-of-Gaussian filtering | σ = 1.0, 2.0, 3.0, 4.0, and 5.0 mm |
| Mathematical transformations | Square, square root, logarithm, and exponential |
| Gradient image | Included |
| Local binary pattern | Three-dimensional LBP, radius 1 voxel, 26 neighboring voxels |
| Feature classes | First-order, shape, GLCM, GLDM, GLRLM, GLSZM, and NGTDM |
| Feature count | 1,874 features: 14 shape features plus 93 nonshape features from each of 20 image types |
| Extraction software | Numericon Research Platform; Python 3.8; PyRadiomics 3.0.1; SimpleITK 2.2.1 |
| IBSI compliance | Feature definitions and preprocessing settings cross-checked against IBSI definitions |
| Mask quality control | All automated masks visually reviewed by two cardiovascular radiologists blinded to invasive FFR; incorrect vessel-wall or nonfat inclusion was corrected before extraction |
| Data export | Feature matrix, parameter file, training-set scaling parameters, patient identifiers, and software versions archived with the analysis output |

Abbreviations: CCTA, coronary computed tomography angiography; CT, computed tomography; FFR, fractional flow reserve; GLCM, gray-level co-occurrence matrix; GLDM, gray-level dependence matrix; GLRLM, gray-level run-length matrix; GLSZM, gray-level size-zone matrix; HU, Hounsfield unit; IBSI, Image Biomarker Standardisation Initiative; LAD, left anterior descending artery; LBP, local binary pattern; LCx, left circumflex artery; NGTDM, neighboring gray-tone difference matrix; PCAT, pericoronary adipose tissue; RCA, right coronary artery; ROI, region of interest; SD, standard deviation.

Wavelet notation: H, high-pass; L, low-pass.

Supplementary Table S4. Patient-level training–validation composition

| Variable | Overall cohort | Training cohort | Validation cohort |
| --- | --- | --- | --- |
| Patients, n | 81 | 57 | 24 |
| Target vessels, n | 107 | 75 | 32 |
| Patients contributing 1 vessel, n (%) | 59 (72.8) | 43 (75.4) | 16 (66.7) |
| Patients contributing 2 vessels, n (%) | 18 (22.2) | 10 (17.5) | 8 (33.3) |
| Patients contributing 3 vessels, n (%) | 4 (4.9) | 4 (7.0) | 0 |
| FFR-positive vessels, n | 47 | 33 | 14 |
| FFR-negative vessels, n | 60 | 42 | 18 |
| Patients with ≥1 FFR-positive vessel, n | 40 | 28 | 12 |
| Patients with all target vessels FFR >0.80, n | 41 | 29 | 12 |
| Vessels per patient, mean ± SD | 1.32 ± 0.57 | 1.32 ± 0.60 | 1.33 ± 0.48 |
| Vessels per patient, median (IQR) | 1 (1–2) | 1 (1–1) | 1 (1–2) |
| Patients appearing in both cohorts, n | 0 | NA | NA |

Abbreviations: FFR, fractional flow reserve; IQR, interquartile range; NA, not applicable; SD, standard deviation.

Supplementary Table S5. Feature-selection workflow and Radscore formula

Panel A. Feature-selection workflow

| Step | Dataset used | Method/parameter | Features retained, n |
| --- | --- | --- | --- |
| Initial extraction | Training cohort only for model development | 1,874 features extracted per vessel | 1,874 |
| Nonfinite/missing-value assessment | Training cohort | No nonfinite values; features with >10% unavailable values would be excluded and remaining values imputed using the training median | 1,874 |
| Variance assessment | Training cohort | Zero-variance features excluded | 1,874 |
| Z-score standardization | Training cohort | Mean and SD estimated in training data; stored parameters applied to validation data | 1,874 |
| Correlation filtering | Training cohort | Pearson correlation; for each pair with \|r\| ≥0.90, the feature with the larger mean absolute correlation was removed | 450 |
| LASSO logistic regression | Training cohort | Binomial logistic LASSO; α = 1.0; outcome coded 1 for invasive FFR ≤0.80 | 3 |
| λ sequence | Training cohort | 100 log-spaced values from λmax to 0.0001 × λmax | — |
| Cross-validation | Training cohort | Ten folds grouped by patient identifier; deviance loss; seed 20260825 | 3 |
| λ selection rule | Training cohort | Largest λ within one standard error of the minimum cross-validated deviance | 3 |
| λ minimum | Training cohort | λmin = 0.019 | — |
| Selected λ | Training cohort | λ1SE = 0.052 | 3 |
| Final feature set | Training cohort | Nonzero coefficients at λ1SE | 3 |
| Classifier fitting | Training cohort | The LASSO linear predictor at λ1SE was retained without post-selection coefficient refitting | 3 |
| Validation | Validation cohort | Frozen feature list, scaling parameters, coefficients, intercept, and cutoff applied without refitting | 3 |
| Software | Primary model development and cluster-adjusted analyses | R 4.3.3; glmnet 4.1-8; pROC 1.18.5; geepack 1.3-12; lme4 1.1-35.3; rsample 1.2.1; tidymodels 1.2.0 | — |

Panel B. Final Radscore formula

| Feature | Image/filter type | Training mean (raw scale) | Training SD (raw scale) | LASSO coefficient |
| --- | --- | --- | --- | --- |
| Intercept | — | — | — | −0.238 |
| original_gldm_SmallDependenceLowGrayLevelEmphasis | Original | 0.0318 | 0.0124 | 0.421 |
| exponential_firstorder_Minimum | Exponential | 0.0247 | 0.0091 | 0.683 |
| wavelet-HHH_glszm_GrayLevelNonUniformity | Wavelet-HHH | 37.62 | 14.85 | −0.512 |

Radscore = −0.238 + 0.421Z₁ + 0.683Z₂ − 0.512Z₃

Z₁ = (original_gldm_SmallDependenceLowGrayLevelEmphasis − 0.0318)/0.0124; Z₂ = (exponential_firstorder_Minimum − 0.0247)/0.0091; Z₃ = (wavelet-HHH_glszm_GrayLevelNonUniformity − 37.62)/14.85.

Abbreviations: FFR, fractional flow reserve; GLDM, gray-level dependence matrix; GLSZM, gray-level size-zone matrix; LASSO, least absolute shrinkage and selection operator; SD, standard deviation; Radscore, radiomics score.

Supplementary Table S6. Candidate classifier comparison

| Classifier | Software/engine | Main hyperparameters | Patient-grouped nested-CV AUC (95% cluster-bootstrap CI) | Accuracy (95% cluster-bootstrap CI) | Selected |
| --- | --- | --- | --- | --- | --- |
| LASSO logistic regression | glmnet 4.1-8 | α = 1.0; λ searched over 100 log-spaced values from λmax to 0.0001 × λmax; λ1SE selected in grouped inner CV | 0.791 (0.671–0.879) | 0.773 (0.666–0.854) | Yes |
| Linear support vector machine | kernlab 0.9-32 | C searched from 2⁻⁵ to 2⁵; selected C = 1.0 | 0.782 (0.657–0.875) | 0.747 (0.635–0.838) | No |
| Radial-basis-function support vector machine | kernlab 0.9-32 | C searched from 2⁻⁵ to 2⁵; selected C = 1.0; γ estimated from the training data within each inner fold | 0.775 (0.644–0.872) | 0.747 (0.632–0.838) | No |
| Random forest | ranger 0.16.0 | 500 trees; mtry = 1–3; minimum node size = 3, 5, or 10; selected mtry = 2 and node size = 5 | 0.751 (0.615–0.854) | 0.733 (0.616–0.829) | No |
| Gradient boosting | xgboost 1.7.8.1 | 50–300 trees; maximum depth = 1–3; learning rate = 0.01–0.10; selected values = 100, 2, and 0.05, respectively | 0.764 (0.632–0.861) | 0.747 (0.629–0.840) | No |
| K-nearest neighbors | kknn 1.3.1 | k = 3, 5, 7, or 9; selected k = 7; predictors standardized within each analysis fold | 0.711 (0.562–0.827) | 0.680 (0.557–0.781) | No |
| Decision tree | rpart 4.1.23 | Maximum depth = 1–5; minimum terminal node size = 3–10; selected depth = 3 and node size = 5 | 0.668 (0.514–0.794) | 0.653 (0.527–0.758) | No |

Abbreviations: AUC, area under the receiver operating characteristic curve; CI, confidence interval; CV, cross-validation; LASSO, least absolute shrinkage and selection operator; Radscore, radiomics score.

Supplementary Table S7. Detailed diagnostic classification results

| Cohort | Model | TP | FN | FP | TN | AUC (95% patient-cluster bootstrap CI) | Accuracy, % (95% cluster-bootstrap CI) | Sensitivity, % (95% cluster-bootstrap CI) | Specificity, % (95% cluster-bootstrap CI) | PPV, % (95% cluster-bootstrap CI) | NPV, % (95% cluster-bootstrap CI) |
| --- | --- | --- | --- | --- | --- | --- | --- | --- | --- | --- | --- |
| Training | DS | 24 | 9 | 16 | 26 | 0.678 (0.550–0.798) | 66.7 (55.3–77.6) | 72.7 (54.8–87.5) | 61.9 (47.6–75.9) | 60.0 (43.3–75.4) | 74.3 (60.4–87.5) |
| Training | CT-FFR | 29 | 4 | 13 | 29 | 0.781 (0.668–0.881) | 77.3 (68.1–86.1) | 87.9 (75.0–97.3) | 69.0 (55.8–82.1) | 69.0 (55.3–81.8) | 87.9 (76.7–97.1) |
| Training | FAI | 20 | 13 | 14 | 28 | 0.643 (0.501–0.773) | 64.0 (52.2–75.0) | 60.6 (40.6–77.8) | 66.7 (52.3–80.4) | 58.8 (40.0–75.6) | 68.3 (54.3–81.4) |
| Training | Radscore | 21 | 12 | 5 | 37 | 0.789 (0.667–0.890) | 77.3 (67.6–86.3) | 63.6 (44.4–80.5) | 88.1 (77.1–97.4) | 80.8 (62.5–95.2) | 75.5 (64.8–85.7) |
| Training | DS + Radscore | 27 | 6 | 10 | 32 | 0.844 (0.742–0.924) | 78.7 (68.5–87.7) | 81.8 (66.7–94.1) | 76.2 (63.0–88.1) | 73.0 (57.6–86.5) | 84.2 (71.8–94.7) |
| Training | CT-FFR + Radscore | 26 | 7 | 5 | 37 | 0.854 (0.754–0.930) | 84.0 (75.3–91.7) | 78.8 (62.9–92.1) | 88.1 (77.3–97.4) | 83.9 (68.8–96.0) | 84.1 (74.4–93.8) |
| Training | FAI + Radscore | 25 | 8 | 8 | 34 | 0.811 (0.692–0.905) | 78.7 (67.7–88.1) | 75.8 (58.6–89.7) | 81.0 (68.2–92.2) | 75.8 (59.1–89.7) | 81.0 (68.4–91.9) |
| Validation | DS | 10 | 4 | 8 | 10 | 0.619 (0.412–0.801) | 62.5 (44.4–80.0) | 71.4 (46.7–92.9) | 55.6 (33.3–77.8) | 55.6 (33.3–76.5) | 71.4 (46.2–93.3) |
| Validation | CT-FFR | 11 | 3 | 5 | 13 | 0.762 (0.561–0.914) | 75.0 (58.1–90.3) | 78.6 (57.1–100.0) | 72.2 (50.0–91.7) | 68.8 (46.2–90.0) | 81.3 (60.0–100.0) |
| Validation | FAI | 7 | 7 | 7 | 11 | 0.585 (0.347–0.797) | 56.3 (40.6–72.7) | 50.0 (23.8–77.8) | 61.1 (37.5–83.3) | 50.0 (26.7–72.7) | 61.1 (40.0–84.2) |
| Validation | Radscore | 8 | 6 | 4 | 14 | 0.684 (0.451–0.873) | 68.8 (51.5–85.7) | 57.1 (26.7–85.7) | 77.8 (57.1–94.7) | 66.7 (37.5–91.7) | 70.0 (50.0–90.0) |
| Validation | DS + Radscore | 10 | 4 | 5 | 13 | 0.812 (0.609–0.944) | 71.9 (52.8–89.7) | 71.4 (46.2–92.9) | 72.2 (50.0–92.9) | 66.7 (40.0–90.0) | 76.5 (53.8–94.4) |
| Validation | CT-FFR + Radscore | 7 | 7 | 2 | 16 | 0.879 (0.694–0.981) | 71.9 (53.3–88.9) | 50.0 (20.0–80.0) | 88.9 (72.2–100.0) | 77.8 (42.9–100.0) | 69.6 (50.0–89.5) |
| Validation | FAI + Radscore | 8 | 6 | 5 | 13 | 0.710 (0.478–0.886) | 65.6 (45.7–84.4) | 57.1 (26.7–86.7) | 72.2 (50.0–92.9) | 61.5 (33.3–86.7) | 68.4 (45.0–90.0) |

Abbreviations: AUC, area under the receiver operating characteristic curve; CI, confidence interval; CT-FFR, computed tomography-derived fractional flow reserve; DS, diameter stenosis; FAI, fat attenuation index; FN, false negative; FP, false positive; NPV, negative predictive value; PPV, positive predictive value; Radscore, radiomics score; TN, true negative; TP, true positive.

Supplementary Table S8. Lesion–ROI concordance and ROI-restricted analysis

Panel A. Concordance between lesion location and the fixed proximal FAI region

| Target vessel | Total lesions | Completely within ROI | Partly within ROI | Outside ROI | Location unavailable |
| --- | --- | --- | --- | --- | --- |
| LAD | 71 | 52 | 9 | 10 | 0 |
| LCx | 13 | 10 | 1 | 2 | 0 |
| RCA | 23 | 21 | 1 | 1 | 0 |
| Overall | 107 | 83 | 11 | 13 | 0 |

Panel B. Sensitivity analysis restricted to lesions completely within the fixed proximal FAI region

| Cohort | Model | Vessels, n | FFR-positive vessels, n | AUC (95% patient-cluster bootstrap CI) | Sensitivity, % (95% cluster-bootstrap CI) | Specificity, % (95% cluster-bootstrap CI) |
| --- | --- | --- | --- | --- | --- | --- |
| Training | DS | 58 | 27 | 0.701 (0.565–0.818) | 74.1 (55.6–88.0) | 61.3 (44.2–75.5) |
| Training | CT-FFR | 58 | 27 | 0.801 (0.683–0.895) | 88.9 (71.9–96.1) | 71.0 (53.6–83.8) |
| Training | FAI | 58 | 27 | 0.671 (0.526–0.794) | 63.0 (44.2–78.5) | 64.5 (47.5–78.7) |
| Training | Radscore | 58 | 27 | 0.812 (0.688–0.902) | 66.7 (47.8–81.5) | 87.1 (70.8–95.0) |
| Training | DS + Radscore | 58 | 27 | 0.861 (0.757–0.932) | 81.5 (63.1–91.8) | 77.4 (60.0–88.7) |
| Training | CT-FFR + Radscore | 58 | 27 | 0.881 (0.786–0.944) | 85.2 (67.5–94.1) | 90.3 (74.9–97.0) |
| Training | FAI + Radscore | 58 | 27 | 0.829 (0.712–0.911) | 77.8 (58.8–89.8) | 80.6 (63.4–90.9) |
| Validation | DS | 25 | 11 | 0.665 (0.405–0.859) | 72.7 (38.9–92.1) | 64.3 (35.6–85.6) |
| Validation | CT-FFR | 25 | 11 | 0.781 (0.573–0.920) | 81.8 (47.8–96.1) | 71.4 (40.6–89.9) |
| Validation | FAI | 25 | 11 | 0.622 (0.361–0.825) | 54.5 (24.6–81.9) | 64.3 (35.6–85.6) |
| Validation | Radscore | 25 | 11 | 0.754 (0.515–0.904) | 63.6 (31.6–86.8) | 85.7 (55.3–96.5) |
| Validation | DS + Radscore | 25 | 11 | 0.834 (0.627–0.947) | 81.8 (47.8–96.1) | 71.4 (40.6–89.9) |
| Validation | CT-FFR + Radscore | 25 | 11 | 0.897 (0.717–0.978) | 63.6 (31.6–86.8) | 92.9 (64.2–99.2) |
| Validation | FAI + Radscore | 25 | 11 | 0.763 (0.529–0.911) | 72.7 (38.9–92.1) | 78.6 (48.8–93.1) |

Abbreviations: AUC, area under the receiver operating characteristic curve; CI, confidence interval; CT-FFR, computed tomography-derived fractional flow reserve; DS, diameter stenosis; FAI, fat attenuation index; FFR, fractional flow reserve; LAD, left anterior descending artery; LCx, left circumflex artery; RCA, right coronary artery; Radscore, radiomics score; ROI, region of interest.
